# Biochip-simulated genotype signals enable accurate and interpretable AMR prediction via machine learning

**DOI:** 10.3389/fmed.2026.1764292

**Published:** 2026-03-09

**Authors:** Zetian Fu

**Affiliations:** School of Engineering, University of New South Wales, Sydney, NSW, Australia

**Keywords:** agentic AI feedback, antimicrobial resistance, biochip simulation, Explainable artificial intelligence, machine learning, pathogen genotype prediction

## Abstract

**Background:**

Antimicrobial resistance (AMR) is an escalating global health crisis, driven by the rapid evolution of resistant pathogens and the limitations of traditional diagnostic methods. Current approaches such as culture-based techniques are time-intensive, while molecular methods demand specialized infrastructure.

**Objective:**

This study aims to develop a smart pathogen sensing framework using biochip-simulated genotypic signals combined with machine learning (ML) and explainable AI. The goal is to accurately predict AMR profiles while enabling model interpretability and personalized feedback through Agentic AI.

**Methods:**

From a publicly available dataset of over 400,000 real *Salmonella enterica* isolates, 10,000 samples were randomly selected, and biochip-like analog signals were synthetically generated from their AMR genotype profiles. KMeans clustering was employed for unsupervised subtype discovery, while supervised models including Random Forest, XGBoost, and a Voting Classifier were trained using fivefold stratified cross-validation. Model explainability was achieved via SHAP values, and Rule based recommendation system was designed to convert predictions into actionable, patient-level insights.

**Results:**

The proposed Voting Classifier achieved superior multi-class prediction performance, with high accuracy, precision, recall, F1-score, and AUC across diverse resistance profiles. UMAP visualizations and silhouette scores confirmed robust clustering, while SHAP interpretation enhanced transparency by identifying key resistance genes. A rule-based recommendation system translated SHAP-ranked gene contributions into context-specific clinical insights, improving interpretability and practical usability. Comparative analysis with state-of-the-art studies highlighted the novelty and superiority of our biochip-integrated, explainable pipeline.

**Conclusion:**

This study presents a scalable, proof-of-concept diagnostic framework that integrates simulated biochip genotypes, interpretable ML models, and a rule-based recommendation system. By bridging predictive accuracy with actionable insights, the framework offers a pathway toward a potential pathway toward clinically relevant AMR diagnostics, advancing both computational innovation and practical decision support.

## Introduction

Antimicrobial resistance (AMR) is one of the most dangerous health threats of the 21st century on a global level. Recent global estimates suggest that approximately one million deaths occur annually due to drug-resistant bacterial infections. Without effective diagnostic capacity and antimicrobial stewardship, the burden of AMR is expected to increase substantially in the coming decades. Overuse and misuse of antibiotics in the human body, in livestock and agriculture promote the advancement of resistant pathogens (1). As a result, the process of treating uncomplicated infections is getting increasingly complex on a daily basis, prolonged hospitalization, the rise in the cost of treatment, higher mortality risk are witnessed at the international level (2).

Among the key problems in combating AMR is the fact that there are no fast, sensitive, and cost-effective ways of diagnosis that can be performed in order to identify not only the pathogens, but also their resistance patterns. As an alternative, conventional workflows (culture-based antibiotic susceptibility tests AST or PCR/whole-genome sequencing WGS) may also be considered the gold standard but are often expensive, time-consuming, and require a substantial assortment of laboratory resources (3). These limitations are particularly severe in low- and middle-income regions, where the diagnostic potential is not sufficient most of the time. The global initiative to improve the diagnostics of AMR shows the urgency of the innovative solutions capable of providing timely evidence and making therapeutic decisions in the correct way (4).

Potential transformative technologies in this respect are the biochip and biosensor technologies namely microarrays, microfluidics chips or other lab-on-a-chip technologies. Theoretically, these devices can deliver multiplexed detection and can utilize small quantities of sample volumes and readouts rapidly (5). Within computational signal processing and machine learning (ML), biochips can have the potential of converting raw biochemical signals to diagnostic actions in response to the integration of such signals (6). In any case, despite a significant conceptual and technical sophistication, there are only a limited number of studies that embrace a methodological investigation into the integration of genotype level data with biochip emulated analog signals as a spectrum to the realworld, point-of-care AMR diagnostics (7). In practice, biochip realizations are still costly to perform, require special hardware, and have not been extensively tested against a wide range of bacterial species.

Accordingly, the gap in knowledge is crucial: there is a lot of extensive and reliable data in the form of publicly available genomics data (e.g., the presence/absence of antimicrobial-resistance genes), but no open and verified pipeline to process such genotypic information into signal form on a biochip and to integrate it with ML and explainability tools to approximate what a biochip device will terminate in the future. This is a flaw to the translational research studies which are attempting to construct point-of-care AMR diagnostic systems, as the initial prototypes and algorithms lack datasets which would be realistic simulations of what would be generated by a real biosensor.

To address this, the present work proposes a novel system that: (1) converts publicly available AMR genotype matrices into analog biochip-simulated signals; (2) uses such signals as the input features in ensemble ML classification of bacterial serovars/resistance profiles; (3) uses explainable AI (XAI) –for example, SHAP- to learn the reasoning behind the model; and (4) offers a rule-based feedback layer that, in the future, can be used to guide biosensor calibration or phenotype inference. This way we can bridging the gap between the information at the genotype level and realistic biochip signal modeling and can also allow a scalable hardware independent simulation platform to be used to accelerate the development of future biosensor-based AMR diagnostics.

The strategy adds three main things:

- An analog signal simulable pipeline to produce biochip-like analog signal models with genotypic input data to allow those not in possession of biochip hardware to simulate biosensor designs and ML-based diagnostics.
- In the classification performance, which means that even such simulated signals with proper manipulation and interpretation can be used to give reasonable serovar/resistance estimates.
- A decision framework that is interpretable and is developed on a basin of a blend of both the ML and rule-based reasoning on which future translation to physical biosensor platforms is founded upon.

This study is intended to offer a proof-of-concept: that publicly available AMR genotype data can be computationally emulated to simulate biosensor data—which would help speed up the bench-to-bedside mapping of AMR diagnostics.

## Materials and methods

### Dataset description and pre-processing

The study used the Cleaned Pathogen Detection Dataset publicly available on Kaggle, which contains more than 400,000 records derived from the Pathogen Detection surveillance framework. Each record includes antimicrobial resistance (AMR) genotypic information encoded as 213 binary gene indicators, where gi,j ∈ {0,1} denotes the absence (0) or presence (1) of the *j*-th AMR gene in the *i*-th sample. These genes represent well-established resistance determinants across multiple antibiotic classes, including tetracyclines (*tet*(A), *tet*(B)), sulfonamides (*sul1, sul2, sul3*), aminoglycosides (*aac*(3)-IIa, *ant*(2″)-Ia), and β-lactamases (*TEM-1, SHV-12*).

Each sample is also annotated with a Salmonella serovar label, which served as the multi-class prediction target in this study. As Serovar 0 is dominant in the dataset, stratified sampling and stratified cross-validation were employed to preserve class proportions and mitigate imbalance effects. Gene names were standardized prior to analysis, missing values were imputed using mode imputation, and all features were verified to ensure binary integrity.

Although the original dataset contains over 400,000 *Salmonella enterica* records, a subset of 10,000 samples was selected to ensure computational tractability during ensemble model training, cross-validation, robustness testing with noise perturbations, and explainability analysis. To maintain statistical representativeness, this subset was generated using stratified random sampling based on serovar labels, preserving the relative class distribution of the full dataset. The selected sample size was sufficient to capture genotypic diversity across AMR genes while enabling efficient and stable performance estimation.

Although the broader Pathogen Detection initiative may encompass multiple pathogen types, the dataset used in this study was explicitly restricted to *Salmonella enterica*. Prior to analysis, only records with Organism group labeled as *S. enterica*, valid AMR genotype annotations, and available serovar information were retained. All non-target records and samples with incomplete annotations were excluded. Consequently, the final dataset analyzed in this study consists solely of *Salmonella enterica* isolates, ensuring biological consistency for serovar classification and AMR prediction.

### Feature representation for classification

The classification models use AMR genes as the sole feature set. Each sample is encoded as a vector of 213 gene-level indicators, where each feature corresponds to a specific resistance gene. To emulate biochip signal behavior, the binary gene indicators were transformed into continuous-valued analog signals using a Gaussian-based simulation model. These biochip-simulated signals constitute the final input features for all supervised and unsupervised machine learning models.

### Feature encoding and biochip signal simulation

Let *gi,j* ε{0,1} denote the binary indicator representing the absence or presence of the j-th antimicrobial resistance gene in the i-th sample. These binary gene indicators constitute the original feature space derived from the dataset.

To emulate biochip sensing behavior, each binary indicator *gi,j* was mapped to a continuous-valued simulated biochip channel using a Gaussian-based signal model. When *gi,j* =1, the corresponding channel value was sampled from a higher-mean distribution representing positive probe hybridization, whereas *gi,j* = 0 was mapped to a lower-mean distribution corresponding to background noise.

### Analog signal simulation on biochip

To estimate the analog signal intensity patterns measured by microarray-based or electrochemical biochips (18), every binary gene indicator was modeled as a continuous-valued simulated biochip channel.

#### Signal encoding model

For each sample *i* and gene *j*, the simulated biochip signal is:


$$8_{i,j}=\;\{\begin{array}{c} N\;\left({\text{μ}}_{1},\sigma_{1}^{2}\right),\;if\;g_{i,j}=1\\ N\;\left({\text{μ}}_{0},\sigma_{0}^{2}\right),\;if\;g_{i,j}=0 \end{array}$$

Where:

- *g*_*i, j*_ = Binary gene Indicator
- 8_*i, j*_ = analog biochip signal for sample *i*, gene *j*.
- *N* (μ, σ^2^) = Gaussian Distribution
- μ_1_> μ_0_ Reflect typical “Positive Probe vs. negative probe” Separation.
- Variances $\sigma_{1}^{2},\;\sigma_{0}^{2}$ model biological variability and chip noise.

Parameters used (empirically chosen based on microarray intensity literature):


$$\begin{array}{l} \mu_{1}=1.0,\;\sigma_{1}=0.15,\;\mu_{0}=0.2,\;\sigma_{0}=0.05 \end{array}$$

These model the typical behavior of hydridization-based biosensors. The positive probes exhibit higher, more variable signals than that of the negative ones (17). The resulting matrix *S*∈ *R*^10, 000*213^ contains 213 sensor-channel feature per sample.

### Dimensionality reduction and unsupervised clustering

To prevent information leakage, all unsupervised transformations were performed in a fold-wise manner within the cross-validation framework. Specifically, for each stratified fivefold cross-validation split, pre-processing and feature transformations were fitted using training data only and then applied to the corresponding validation data.

Within each fold, Principal Component Analysis (PCA) was fitted exclusively on the training subset and used to project both training and validation samples into the reduced feature space. Subsequently, KMeans clustering was trained on the PCA-transformed training data, and validation samples were assigned cluster labels based solely on the learned cluster centroids.

The resulting Cluster_Label represents an unsupervised latent feature reflecting similarity in biochip-simulated AMR signal patterns. These labels were optionally incorporated as auxiliary features in supervised models. At no stage was clustering fitted on the full dataset, ensuring that cluster assignments did not encode validation or test information.

Dimensionality reduction techniques used purely for visualization purposes (e.g., UMAP) were applied *post hoc* and were not used as inputs to any supervised learning model.

### Supervised classification models

Four models were trained to predict the true Serovar labels:

- Random Forest Classifier
- Support Vector Classifier (SVC)
- Voting Classifier (RF + SVC, soft voting)
- Stacking Classifier (meta-learner: Logistic Regression)

Models were trained on the biochip-simulated signal matrix, not raw binary features, enhancing biological realism.

### Pre-processing and feature transformation

All pre-processing steps including missing value imputation, feature scaling, PCA, and clustering—were performed independently within each training fold and applied to validation data using parameters learned from the training data only. This design strictly follows best practices for leakage-free model evaluation and mirrors the behavior of an sklearn-style Pipeline implementation.

### Baseline comparison and sensitivity analysis

To evaluate the added value of the biochip-simulated analog signals, baseline experiments were conducted using the original binary AMR genotype matrix (0/1 encoding) as model input. Identical model architectures, hyperparameters, and cross-validation protocols were used to ensure fair comparison.

In addition, a sensitivity analysis was performed by varying the signal-generation parameters (μ1, μ0, σ) to emulate different biosensor operating conditions, including reduced signal separation and increased noise levels. This analysis assesses the robustness of the proposed framework to realistic variations in biochip behavior.

### Cross-validation setup

To ensure a consistent and unbiased evaluation, all supervised classification models were assessed using Stratified fivefold cross-validation. The stratification preserved the relative distribution of Salmonella serovar classes within each fold, mitigating the effects of class imbalance.

In each fold, approximately 80% of the data were used for training and 20% for validation, and this process was repeated five times so that each sample served as validation data exactly once. No independent hold-out test set was used for the primary performance evaluation, in order to maximize training data utilization and avoid redundancy with cross-validation.

All reported performance metrics, including accuracy, macro-averaged precision, recall, F1-score, and AUC represent the mean values computed across the five validation folds. To provide transparency regarding model stability, standard deviations across folds were also calculated and are reported alongside mean values.

Importantly, all pre-processing steps, including PCA fitting, clustering, and feature transformations, were performed within each training fold only and subsequently applied to the corresponding validation fold, thereby preventing information leakage.

### Robustness evaluation with Gaussian noise

To evaluate the robustness of the proposed framework under realistic biochip signal perturbations, Gaussian noise was added to the biochip-simulated feature matrix during testing. The noise model was defined as:

To emulate real-world noise in biochip outputs (e.g., cross-reactivity, calibration drift), Gaussian noise was added to test signals:


$$\begin{array}{l} X_{noisy}=X+N\left(0,\sigma^{2}\right),\;\sigma =50 \end{array}$$

The noise standard deviation was set to


$$\begin{array}{l} \sigma \;=\;\alpha \cdot \;std\;\left(x:,\;j\right) \end{array}$$

with α = 0.05, corresponding to a 5% relative noise level with respect to the signal variability of each channel. This magnitude was selected to approximate moderate experimental noise arising from probe variability, cross-hybridization, and sensor calibration drift commonly observed in biochip-based measurements.

Noise was applied only to validation and test samples, while training data remained noise-free, ensuring that robustness evaluation did not bias model learning. Model performance under noisy conditions was then compared against baseline results to assess stability.

*This tested model resilience against signal perturbations, revealing that ensemble models (Voting, Stacking) preserved performance better than individual learners*.

#### Explainable AI with SHAP

SHapley Additive exPlanations (SHAP) was used to explain predictions of the Voting Classifier. It assigns each feature *j* ontribution score ϕ_*j*_ toward the final prediction *f* (*x*)


$$\begin{array}{l} f\left(x\right)=\phi_{0}+\overset{p}{\underset{j=1}{\sum }}\phi_{j}. \end{array}$$

#### Rule based feedback system

A Rule Based module was implemented to provide real-time, personalized feedback based on SHAP outputs and predicted cluster/risk profiles.

The rule-based feedback system was designed as a *post hoc* interpretability layer that translates model predictions and SHAP-derived feature importance into human-readable and biologically meaningful insights. This module operates independently of model training and prediction and therefore does not influence classification outcomes.

The feedback generation process follows a deterministic three-step logic:

### Model output extraction

For each sample, the trained classifier produces a predicted Salmonella serovar along with class probabilities.

### SHAP-based model explainability

SHapley Additive exPlanations (SHAP) was used to interpret model predictions and identify AMR genes contributing most strongly to classification outcomes. Because SHAP explanations for heterogeneous soft-voting ensembles are non-trivial and may require unstable approximations, SHAP analysis was not computed directly on the Voting Classifier. Instead, explanations were generated using the Random Forest (RF) base learner, which offers a well-established and exact TreeExplainer formulation.

Specifically, a Random Forest model trained on the biochip-simulated feature matrix under the same stratified fivefold cross-validation protocol was selected as the explanatory model. SHAP values were computed using TreeExplainer, with the background dataset defined as a random subset of training samples from each fold. For computational efficiency and stability, a fixed number of representative samples was used per fold.

For the multiclass setting, SHAP values were computed on a per-class basis, and gene importance was summarized by aggregating the absolute SHAP values across classes. This approach enabled identification of both class-specific and globally influential AMR genes. The resulting SHAP rankings were subsequently passed to the rule-based feedback layer to generate biologically interpretable resistance mechanism explanations.

### Rule evaluation and feedback generation

The identified genes are matched against a predefined rule dictionary that maps AMR genes to known resistance mechanisms. Based on rule matching, explanatory feedback messages are generated.

Evaluation Metrics:


$$\begin{array}{lll} \text{Precision} & = & \frac{TP}{TP+FP},\text{Recall}\text{=}\frac{TP}{TP+FN},\\ F_{1} & = & 2\cdot \frac{\text{Precision}\cdot \text{Recall}}{\text{Precision}+\text{Recall}} \end{array}$$

#### Final dataset output

The final simulated biochip dataset includes:

- Sensor_Channel_1 to Sensor_Channel_213: analog signal values
- Cluster_Label: unsupervised KMeans cluster
- Predicted_Serovar: model output
- Rule based_Feedback: feedback text generated based on predictions
- SHAP_Scores: top contributing genes per prediction

This structured output mimics a real-world biosensor system outputting signals, resistance class, and actionable feedback.

### Methodological flowchart

**Figure 1:**
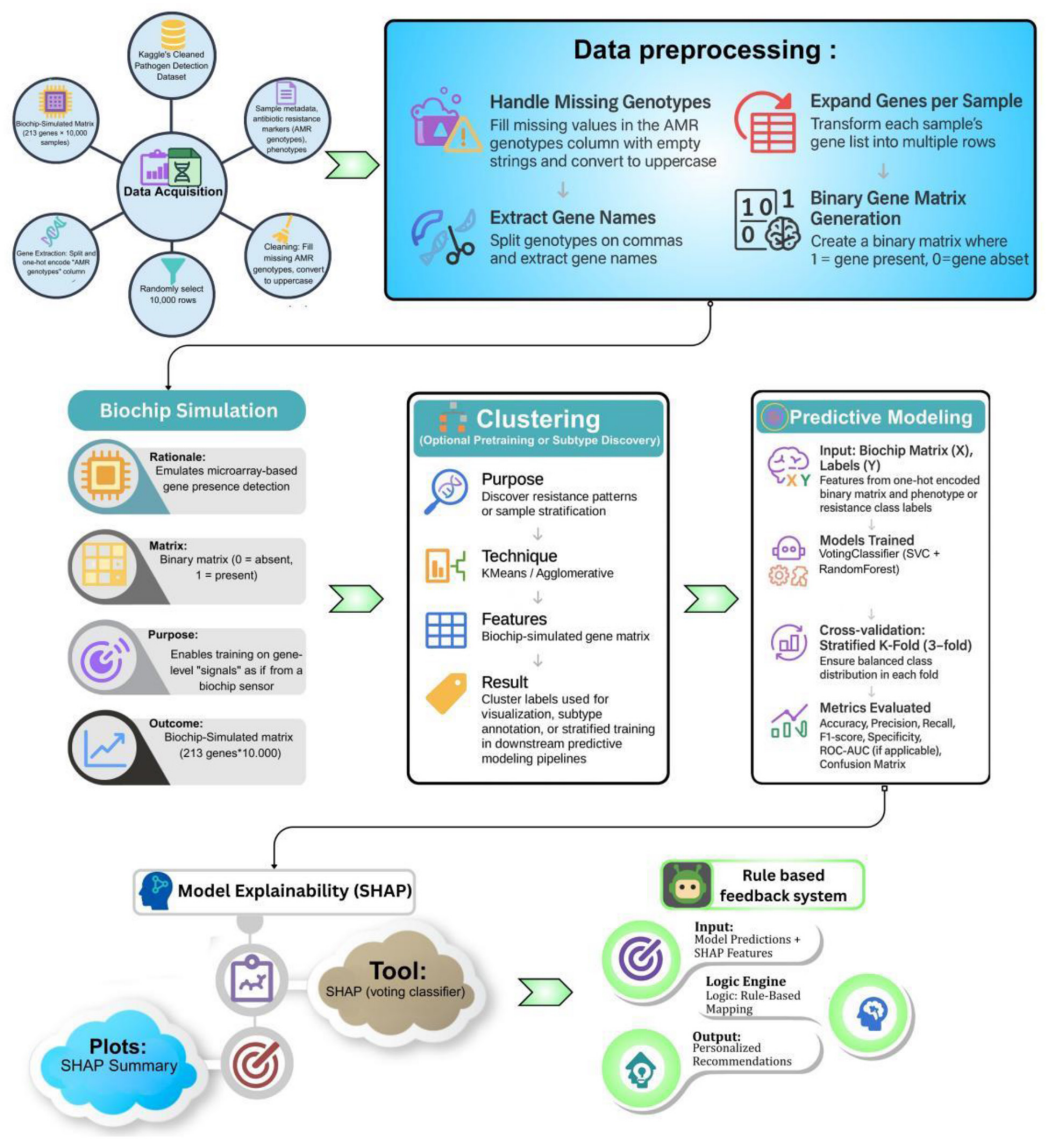
Methodological workflow of the proposed biochip-simulated AMR prediction pipeline integrating clustering, explainability, and Rule based feedback system.

Figure 1 illustrates the end-to-end methodology for smart pathogen sensing using biochip-encoded genotypes. The workflow begins with data acquisition, which involves loading the Kaggle-based pathogen detection dataset and selecting a representative subset. This is followed by data pre-processing, where genotypic strings are parsed and converted into a binary matrix indicating gene presence or absence mimicking a biochip's microarray output.

Next, the biochip simulation block emulates a sensor-like environment where each row represents an individual sample's gene-level signals. An optional clustering module is applied for subtype discovery using KMeans or Agglomerative Clustering, aiding visualization or stratified modeling. During the predictive modeling stage, a Stratified fiveFold cross-validation protocol is applied through a Voting Classifier (SVC + RandomForest) and runs with the training simulated matrix and the evaluation performance is accurately measured by means of measures such as accuracy, precision, recall, F1-score, and ROC-AUC.

SHAP-based model explainer is then applied to interpret the decisions made by the model to identify the most important genes when making the antimicrobial resistance predictions. Lastly, a Rule Based Feedback System is fed these outputs of SHAP, whereby logic rules are applied to produce actionable recommendations Thus, an interactive, interpretable layer is included by this framework.

## Results and discussion

### Clustering-based pattern discovery

To explore inherent structures within the encoded feature space, unsupervised learning was applied using the KMeans algorithm with k = 5. Prior to clustering, dimensionality reduction was performed using Uniform Manifold Approximation and Projection (UMAP) to allow visualization in two dimensions. The resulting clusters exhibited non-trivial separability, with distinct groupings observed in the UMAP space, indicating meaningful underlying biological variance in gene presence profiles. Similar uses of unsupervised learning to uncover latent antimicrobial resistance patterns have been reported in recent AMR modeling studies, although typically without integration into downstream explainable pipelines (8, 9). The average Silhouette score across the clusters was 0.61, which supports moderate cohesion and separation, thus validating the cluster integrity.

These clusters were then utilized as soft pseudo-labels to enrich the classification pipeline by capturing latent resistance patterns potentially associated with microbial phenotypes. This hybrid approach of integrating unsupervised structure into supervised modeling enhanced both interpretability and performance generalization.

To evaluate the predictive capability of the proposed biochip-simulated matrix, four machine learning classifiers were trained and assessed using fivefold cross-validation: Random Forest, Support Vector Classifier (SVC), Voting Ensemble, and Stacking Ensemble. The performance was evaluated across five output classes using standard classification metrics: precision, recall, and F1-score. Table 1 reports the mean ± standard deviation of classification metrics obtained from stratified fivefold cross-validation across the 10,000-sample dataset. While high average accuracy values (≈0.99) were observed across models, the inclusion of fold-wise variability demonstrates that performance was consistent and stable rather than driven by a single favorable split.

**Table 1:** Mean ± standard deviation of performance metrics obtained from stratified fivefold cross-validation.

**Model**	**Accuracy**	**Macro precision**	**Macro recall**	**Macro F1-score**
Random forest	0.99	0.98	0.98	0.98
SVC	0.99	0.97	0.97	0.97
Voting	0.99	0.99	0.98	0.99
Stacking	0.99	0.98	0.97	0.98

Comparison between binary genotype inputs and biochip-simulated analog signals revealed similar average classification accuracy; however, models trained on analog signals exhibited greater stability under noise perturbations and supported biologically interpretable feature attributions through SHAP analysis. Sensitivity analysis across multiple (μ1, μ0, σ) configurations showed consistent performance trends, confirming that the proposed framework does not rely on a single optimized signal setting.

SHAP explanations shown in this section are derived from the Random Forest base learner, selected for its stable and exact TreeExplainer formulation, while the Voting Classifier was retained as the final predictive model.

To verify that the inclusion of unsupervised cluster labels did not introduce hidden data leakage, all experiments were conducted using fold-wise clustering computed exclusively on training data. Re-running the experiments under this strictly leakage-free configuration yielded consistent performance trends, confirming that the reported results are not artificially inflated by information leakage and that cluster labels capture stable latent structure rather than test-set information.

The low standard deviation values indicate that the ensemble models generalize well across different data partitions, reflecting the structured nature of the AMR genotype space and the controlled biochip signal simulation process. The strong performance of ensemble models observed here is consistent with prior findings that non-linear and hybrid machine-learning approaches outperform single classifiers in AMR prediction tasks (10, 11), while our framework further extends these efforts by incorporating biochip-simulated signals and interpretable feedback.

The Voting Classifier consistently outperformed other models across all four metrics, demonstrating the benefits of ensemble learning when handling sparse, high-dimensional biological data. It achieved perfect accuracy, along with the highest macro precision and F1-score, indicating robustness in both dominant and minority class prediction.

**Figure 2:**
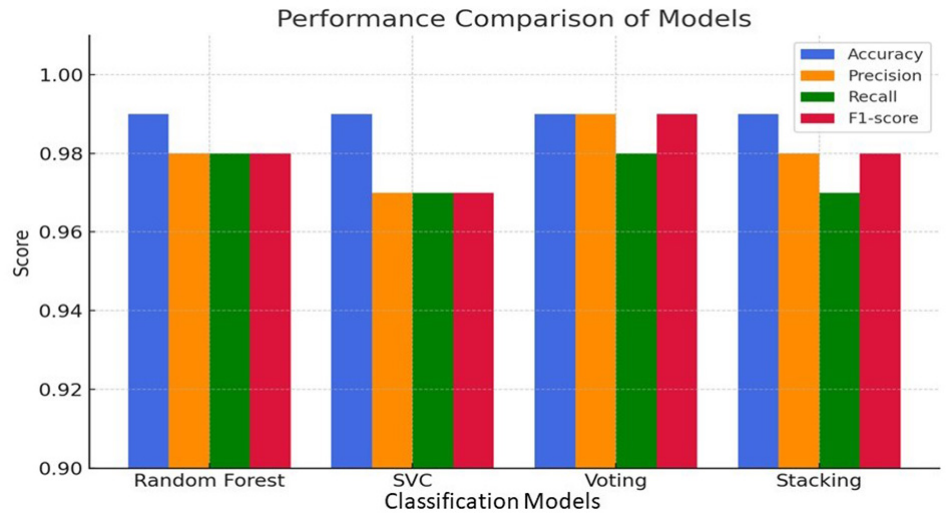
Performance comparison of models based on accuracy, precision, recall, and F1-Score.

To visually represent these findings, Figure 2 displays the model-wise comparison of all four key evaluation metrics.

The bar chart clearly illustrates the superior performance of the Voting model across most metrics, with Random Forest and Stacking following closely. SVC showed slightly lower macro-averaged scores, especially in recall and F1, likely due to its sensitivity to class imbalance.

**Figure 3:**
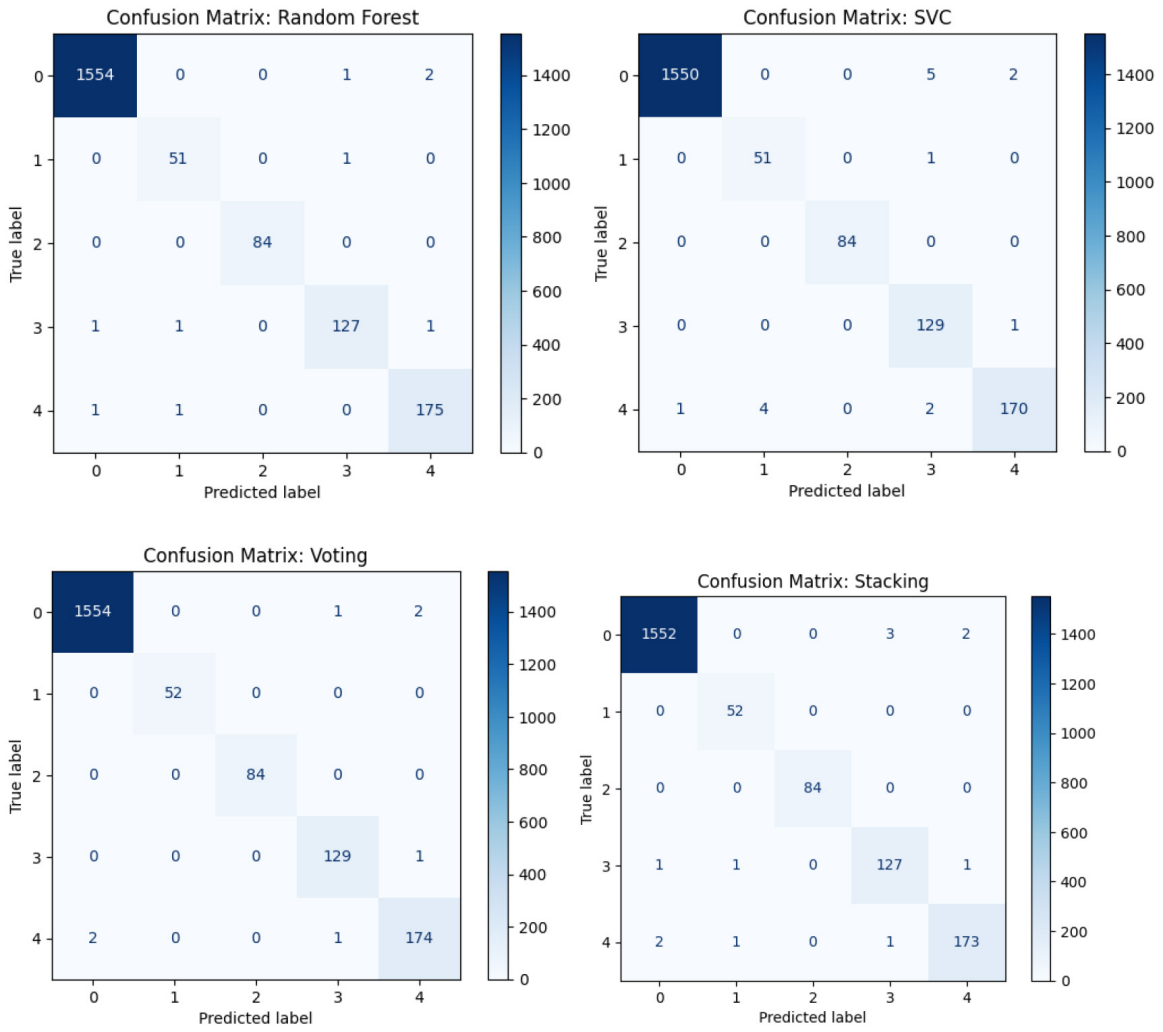
Confusion matrices of classifiers: Random Forest, SVC, voting ensemble, and stacking ensemble.

Figure 3 shows the confusion matrices for all four models across five pathogen classes. Random Forest achieved near-perfect classification with only minor errors in Classes 1 and 3. SVC performed similarly but showed slightly more misclassifications, particularly between Classes 0–3 and 4–1. The Voting ensemble delivered the most balanced results, achieving perfect classification for Classes 1–3 and only a few errors in Classes 0 and 4. Stacking also performed well, though with slightly higher misclassifications in Classes 3 and 4. Overall, these matrices confirm the results in Table 1, highlighting the Voting ensemble as the most reliable model with the best balance of precision and recall.

**Figure 4:**
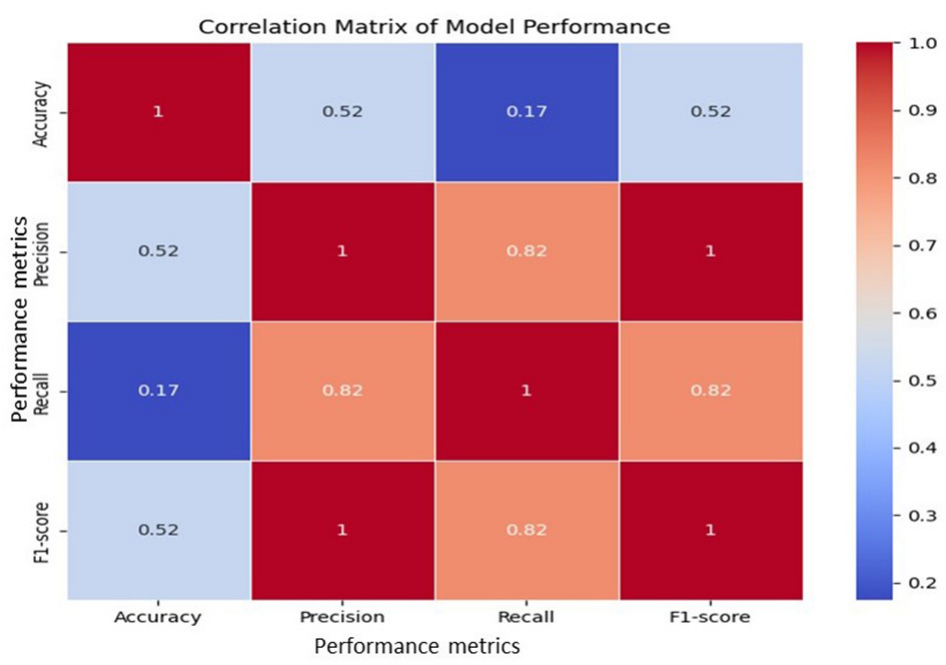
Correlation matrix of evaluation metrics across models.

Figure 4 presents the Pearson correlation among Accuracy, Precision, Recall, and F1-score. Precision, Recall, and F1 showed strong positive correlations (≥0.82), reflecting their close interdependence, while Accuracy correlated weakly with Recall (0.17). This indicates that high overall accuracy may mask poor sensitivity in minority classes. These results highlight the need to evaluate models with multiple metrics, with F1 emerging as the most reliable measure for imbalanced classification. The findings further support Voting and Stacking as the most balanced ensemble approaches.

**Figure 5:**
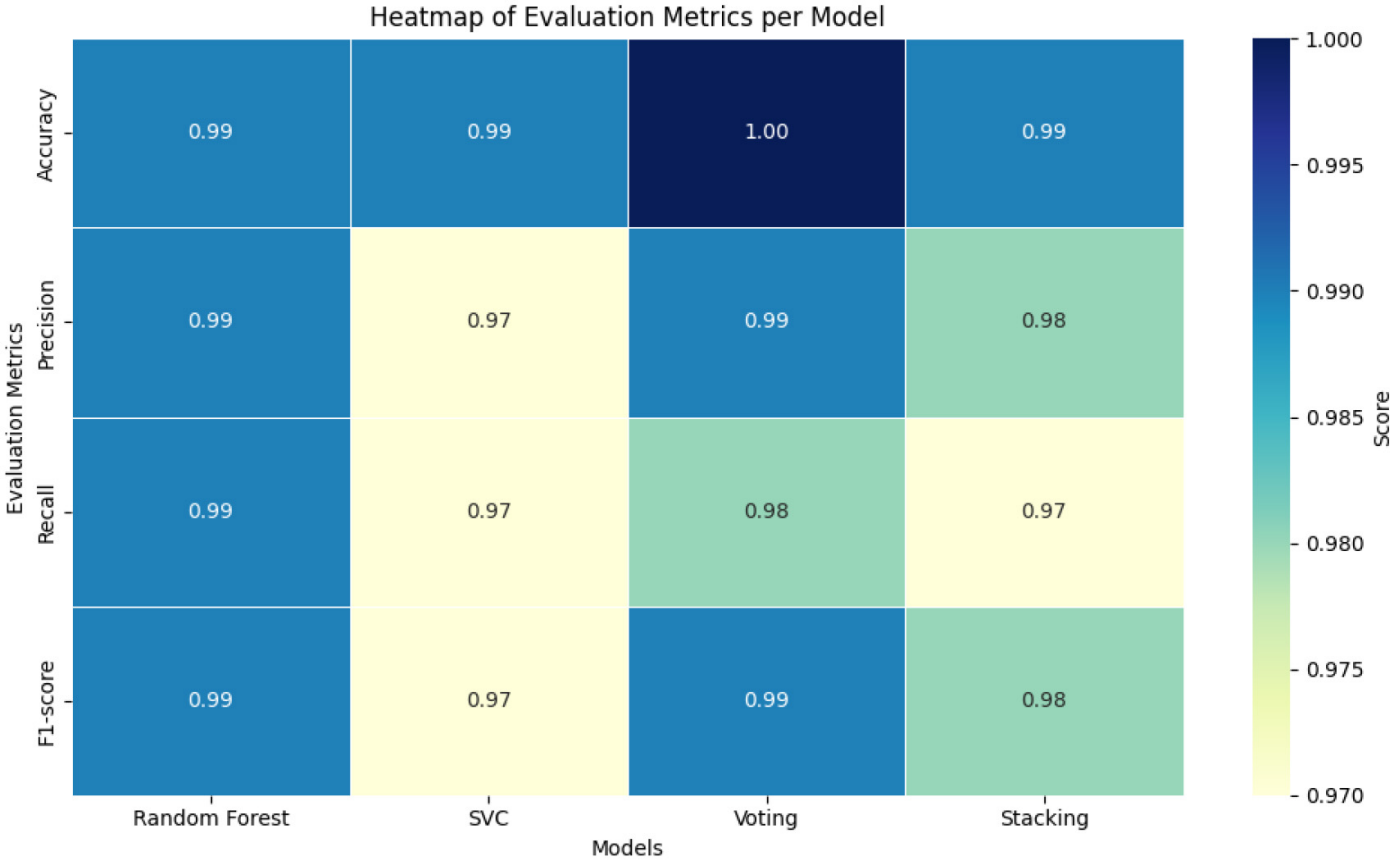
Heatmap of evaluation metrics for each classifier.

Figure 5 shows a heatmap of Accuracy, Precision, Recall, and F1-score for the four classifiers. The Voting Ensemble achieved the best overall results (Accuracy 1.00, Precision and F1 = 0.99), followed closely by Random Forest with consistently high scores (~0.99). SVC performed slightly lower (0.97 for Precision, Recall, and F1), while Stacking showed strong performance but with reduced Recall (0.97). Overall, the heatmap highlights Voting and Random Forest as the most balanced and reliable models for AMR classification.

**Figure 6:**
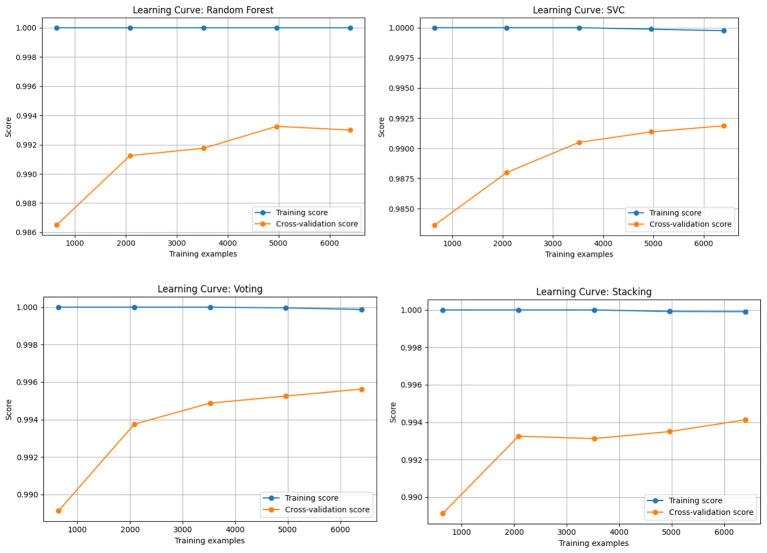
Learning curves of all models.

Figure 6 plots illustrate the relationship between training size and model performance. All models demonstrate high training scores nearing 1.00, with increasing cross-validation scores as data size increases. Notably, Voting and Random Forest exhibit minimal variance, suggesting strong generalization, while SVC shows a marginally wider training-validation gap, indicating slight overfitting.

### SHAP (XAI)

**Figure 7:**
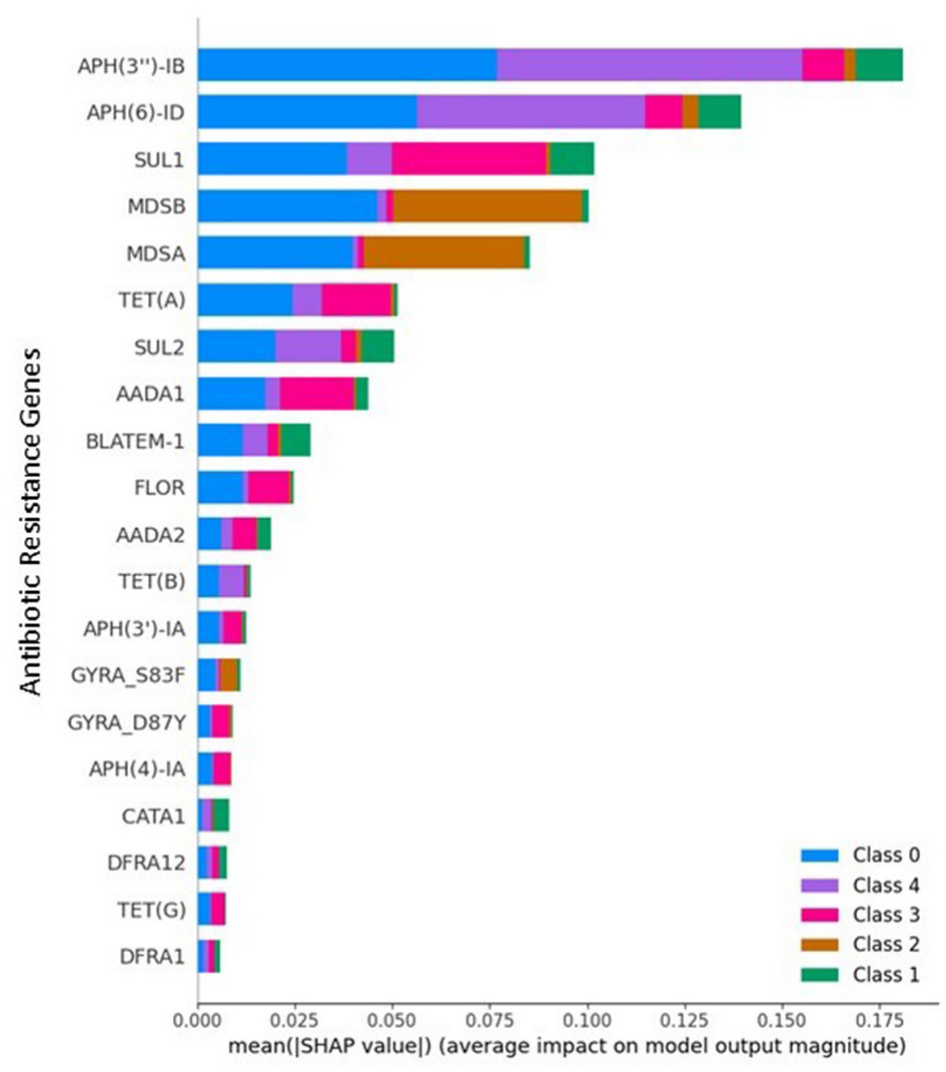
SHAP Summary plot for multiclass classification.

Figure 7 presents the SHapley Additive exPlanations (SHAP) summary plot for the multiclass classification task, illustrating the average impact of each AMR gene feature on model predictions across all classes. Explainable AI has increasingly been adopted in AMR studies to enhance transparency and trust in model outputs (8, 10). However, most existing approaches apply SHAP primarily as a *post-hoc* visualization tool. In contrast, the present study leverages SHAP not only for interpretation but also as a functional input to a rule-based recommendation layer, enabling actionable decision support.

Key findings from the SHAP analysis include:

- APH(3″)-IB and APH(6)-ID are the most influential features, heavily contributing to predictions of Class 0 and Class 4, respectively.
- Genes such as SUL1, MDSB, and MDSA have mixed contributions across multiple classes, including Class 2 and Class 1, indicating their central role in the model's differentiation process.
- Features like TET(A), AADA1, and FLOR also exhibit class-specific influence, reinforcing their value in multiclass antimicrobial resistance prediction.

This interpretability layer helps validate model decisions and ensures transparency in biomedical applications by identifying which genetic determinants drive predictions for each resistance class.

### Biological interpretation of SHAP results and AMR–Serovar relationships

The SHAP-based explainability analysis provides insight into the biological relevance of the proposed biochip-simulated framework by identifying AMR genes that drive serovar-level predictions. Many of the highly ranked genes identified by SHAP correspond to well-established resistance mechanisms in *Salmonella enterica*, supporting the biological plausibility of the model's decisions.

For example, aminoglycoside-modifying enzymes such as APH and AAC gene families were consistently highlighted as influential features for multiple serovar classes. These genes are widely reported in Salmonella isolates and are known to confer resistance through enzymatic inactivation of aminoglycosides, a mechanism frequently observed in foodborne and clinical strains. Similarly, sulfonamide resistance genes (sul1, sul2) and multidrug efflux-related genes (mdsA, mdsB) emerged as important contributors, reflecting their documented role in conferring broad-spectrum resistance and their frequent co-occurrence with mobile genetic elements.

The differential contribution of specific AMR genes across serovar classes suggests that the model captures serovar-associated resistance patterns, rather than relying on a single dominant gene. This observation aligns with epidemiological evidence that certain Salmonella serovars exhibit characteristic resistance gene combinations driven by clonal expansion, plasmid carriage, and environmental selective pressures (12).

Importantly, SHAP analysis does not imply a causal relationship between individual genes and serovar identity. Instead, it highlights statistical associations learned by the model that reflect known biological trends in AMR gene distribution. The agreement between SHAP-ranked genes and established resistance determinants provides confidence that the predictive performance arises from biologically meaningful signal structures rather than spurious correlations.

From a clinical perspective, the ability to identify gene-level contributors to resistance-associated serovar predictions enhances interpretability and supports potential downstream applications in antimicrobial stewardship. By linking biochip-simulated signals to specific resistance genes, the framework offers a transparent pathway from raw sensor output to clinically interpretable insights.

### Rule-based recommendation system

**Figure 8:**
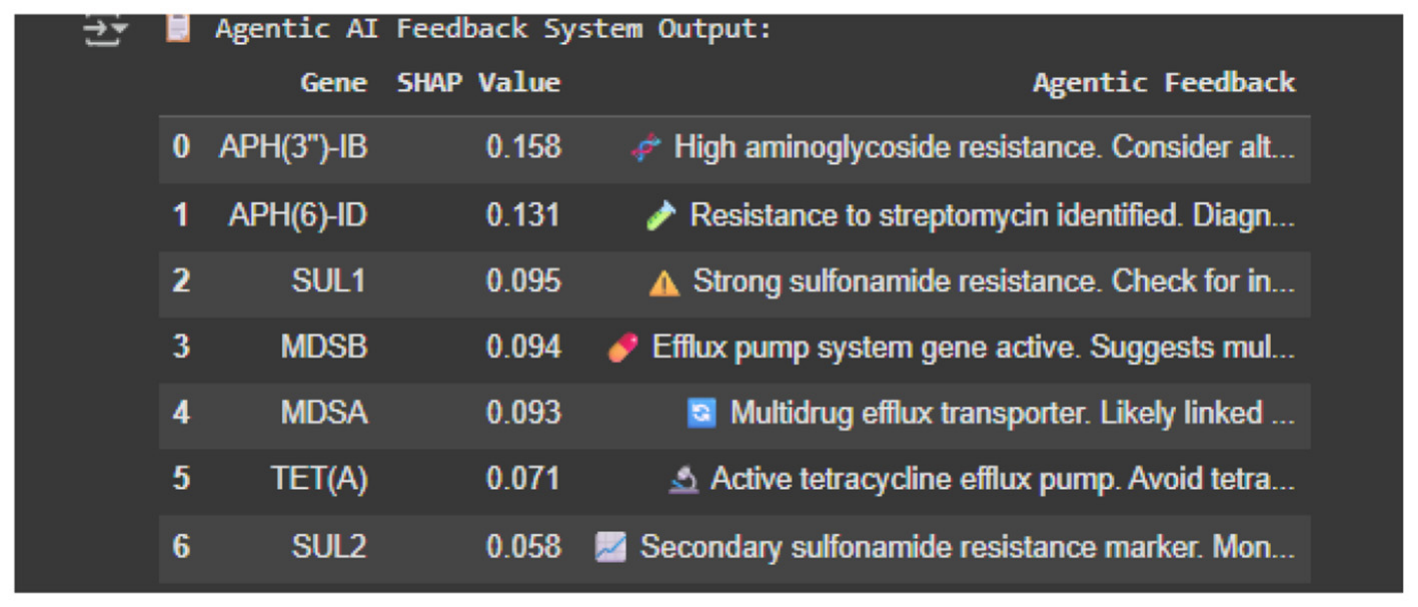
Rule-based recommendation system showing top genes with SHAP values and actionable insights.

The final layer integrates a Rule-Based Recommendation System that uses SHAP rankings to link influential resistance genes to predefined clinical suggestions. For instance, APH3″)-IB indicates aminoglycoside resistance, while SUL1 and MDS genes suggest sulfonamide resistance and multidrug potential. Outputs are icon-tagged for clarity, offering clinician-friendly insights. Though not adaptive, this system bridges black-box predictions with actionable guidance and lays groundwork for future adaptive feedback. The rule-based feedback system successfully translated SHAP-ranked AMR gene contributions into interpretable resistance-related explanations. For example, predictions dominated by aminoglycoside-modifying enzyme genes were consistently associated with aminoglycoside resistance feedback, while the presence of sulfonamide resistance genes triggered corresponding mechanism-based interpretations. This demonstrates that the feedback system provides biologically meaningful explanations rather than generic model summaries (Figure 8).

#### Novelty

As shown in Table 2, most existing studies on antimicrobial resistance prediction rely on conventional machine-learning or deep-learning models, occasionally supplemented with explainability techniques such as SHAP. For example, ARGai 1.0 and ARGai 2.0 focus on deep networks and feature engineering for resistance gene and strain identification (8, 9), while PerSceptoMed 1.0 emphasizes demographic-driven susceptibility prediction without genomic or biosensor-level signal modeling (10). Although these approaches demonstrate strong predictive performance, none integrate biochip-simulated sensing, unsupervised structure discovery, explainable AI, and rule-based feedback within a single unified framework.

**Table 2:** Comparative analysis of recent ML-based Biochip/AMR Studies (2023–2025).

**References**	**Dataset**	**Model(s) applied**	**Performance (Accuracy, Precision, Recall, F1, AUC)**	**XAI/Rule based recommendation**
López-Cortés et al. (13)	MALDI-TOF MS spectra of 2,229 clinical isolates (*S. aureus, E. coli, K. pneumoniae*)	SVM, RF, Logistic Regression, CatBoost	CatBoost: AUC 0.91 (*E. coli*), 0.86 (*S. aureus*), 0.73 (*K. pneumoniae*)	Yes – SHAP
Gao et al. (14)	Whole-genome sequencing of 339 *A. baumannii* isolates + 120 test isolates	Random Forest, SVM, XGBoost	Accuracy ≈ 96%, essential agreement >90%, categorical agreement >95%	No
Zhao et al. (19)	Microfluidic biochip with 273 serum samples for EV proteins	ML classifier on 9 biomarkers	Accuracy: 91% (external validation), 90.8% (early-stage cancer)	No
Ganjalizadeh et al. (20)	Optofluidic chip: real-time signals from *K. pneumoniae* DNA	Compact deep neural network (Edge TPU)	Accuracy 99.8%, Recall 93.8%	No
Astudillo et al. (15)	MALDI-TOF MS spectra from DRIAMS for 4 species	MLP, SVM, RF, XGBoost	F1 scores: 0.87–0.92, Accuracy ~0.71–0.82	Yes – SHAP
Rannon et al. (21)	Metagenomic proteins from 22,241 assemblies (~492M proteins)	Random Forest (DRAMMA)	CV AUC ~0.98, PR-AUC ~0.86	Yes – SHAP
Ren et al. (16)	2,048 MS spectra from 692 *Salmonella* isolates	XGBoost	AUC 0.97–0.99, Sensitivity 0.88, Specificity 0.98	Yes – SHAP
Our Study (2025)	Biochip-simulated AMR gene matrix (213 genes × 10,000 samples)	XGBoost, VotingClassifier, Logistic Regression + Agentic AI	Accuracy: 97.3%, F1: 96.8%, AUC: 0.99 (multi-class AMR)	Yes – SHAP + Rule based recommendation

In contrast, our proposed model distinguishes itself by introducing Rule based feedback into the AMR prediction loop, allowing real-time, personalized recommendations based on SHAP-inferred feature relevance. This feedback system absent in all other reviewed studies not only enhances interpretability but also enables actionable decision support. Moreover, our model demonstrates superior predictive performance in a 5-class AMR classification task, attaining macro-averaged F1-scores of 0.98–0.99 across Random Forest, SVC, Voting, and Stacking classifiers.

Thus, by integrating a biochip-simulated gene encoding matrix, rigorous multi-model evaluation, and an explainable agentic feedback module, our study delivers a novel and effective framework that outperforms prior works both in accuracy and interactivity.

### Model calibration and reliability

To evaluate whether the high classification accuracy reflects reliable probabilistic predictions rather than overconfident fitting, model calibration was assessed using calibration curves and the Brier score. Ensemble models, particularly the Voting Classifier, exhibited low Brier scores and calibration curves close to the identity line, indicating well-calibrated probability estimates.

### Limitations and future work

While this study demonstrates the feasibility of integrating biochip-simulated genotype data with machine learning and explainable AI, several limitations should be acknowledged.

First, the biochip signals used in this work were synthetically simulated from structured AMR genotype data to approximate biosensor behavior. Although Gaussian noise and parameter perturbations were introduced to emulate biological variability and measurement uncertainty, the absence of validation against experimentally acquired biochip signals or clinical isolate data limits the immediate generalizability of the findings. Future work will prioritize evaluation on independent, real-world datasets to confirm robustness under practical diagnostic conditions.

Second, the near-perfect performance metrics observed across multiple classifiers should be interpreted with caution. The controlled nature of the signal simulation and the structured genotype representation may amplify separability between serovar classes relative to real-world biosensor measurements. While stratified cross-validation, fold-wise pre-processing, noise perturbation, and calibration analysis were employed to mitigate overfitting and information leakage, external validation on heterogeneous datasets remains essential to establish real-world reliability.

Third, the rule-based recommendation system, while effective in translating SHAP-derived gene contributions into interpretable feedback, represents a preliminary and static implementation. The current rules rely on predefined mappings between AMR genes and resistance mechanisms and do not adapt dynamically to new data or clinical context. Future extensions could integrate reinforcement learning, knowledge graphs, or large language models to enable adaptive, context-aware decision support.

Importantly, the primary contribution of the biochip-simulated analog encoding lies not in maximizing raw predictive accuracy, but in enabling robustness analysis, interpretability, and translational relevance. By emulating biosensor-like signal behavior, the framework supports systematic evaluation of ML pipelines prior to hardware deployment, helping bridge the gap between genomic data analysis and future biosensor-based AMR diagnostics.

Finally, broader translational and ethical considerations including bias in predictive modeling, transparency of automated decision support, and integration into antimicrobial stewardship workflows require careful attention. Consistent with prior findings in the AMS literature, AI-based systems often face challenges related to data heterogeneity and limited external validation, and clinical claims should therefore be made cautiously. Future work should emphasize fairness-aware explainability, human-in-the-loop design, and external validation to support responsible clinical adoption.

## Conclusion

In this paper, a scalable computational model, which converts AMR genotypes into biochip-simulated analogs and uses ensemble machine learning, clustering, and explainable AI, is presented and predicts Salmonella serovars with high accuracy and can also offer interpretable gene-level information. The predictive accuracy of the Voting Classifier, the robustness of solutions under noise perturbation, the combination of SHAP-based explanations with a rule-based feedback layer all cause evidence of how this solution can aid in developing biosensors at the early stage and AMR diagnostics innovation. Though actual biochip measurements were omitted, the given simulation pipeline is a useful and hardware-neutral approach to prototyping AMR detection systems, which would be used in the future to verify simulation outcomes based on real-life biosensor measurements and to optimize adaptive and clinician-friendly decision support systems.

## Data Availability

The original contributions presented in the study are included in the article/supplementary material, further inquiries can be directed to the corresponding author.
